# A method to include exclusive heavy vector-meson production data at small *x* in global parton analyses

**DOI:** 10.1140/epjc/s10052-025-14142-9

**Published:** 2025-04-18

**Authors:** C. A. Flett, A. D. Martin, M. G. Ryskin, T. Teubner

**Affiliations:** 1 Université Paris-Saclay, CNRS, IJCLab, 91405 Orsay, France; 2 Institute for Particle Physics Phenomenology, Durham University, Durham, DH1 3LE UK; 3 Petersburg Nuclear Physics Institute, NRC Kurchatov Institute, Gatchina, St. Petersburg, 188300 Russia; 4 Department of Mathematical Sciences, University of Liverpool, Liverpool, L69 3BX UK

## Abstract

We propose a method which allows the inclusion of exclusive heavy vector-meson production data at low *x* in future global parton analyses. As an example we perform a study within xFitter to determine the gluon parton distribution function (PDF) at next-to-leading order (NLO) at moderate-to-low *x* using the measurements of exclusive $J/\psi$ production in *ep* and *pp* collisions from HERA and LHC. We further study the constraints from the corresponding $\Upsilon$ production process. We finish by discussing the possible effects at next-to-next-to-leading order (NNLO) through incorporation of a *K* factor for the exclusive heavy vector-meson coefficient function at NLO.

## Introduction

The precision of the parton distributions of the proton is well established through global analyses [1–3], provided the resolution scale $Q^2$ is not too low and the momentum fraction *x* remains within a moderate range, neither too small nor too large. As *x* decreases, particularly at lower scales, the uncertainty in these distributions increases significantly. This increase in uncertainty is due to the lack of experimental data directly probing this region.

The experiments at the Large Hadron Collider (LHC) are capable of particle detection and reconstruction over a wide rapidity range. Notably, various measurements of the differential cross sections for exclusive heavy vector mesons such as $J/\psi$ and $\Upsilon$ [4–6] have allowed for the determination of the gluon Parton Distribution Function (PDF) down to $x \sim 3\times 10^{-6}$ at factorisation scales $\mu _F = m_q$, where $q = c,b$.

**Fig. 1:**
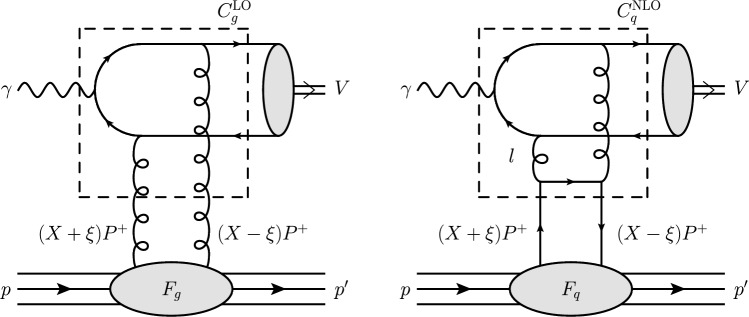
(Left) LO contribution to $\gamma p \rightarrow V +p$, where the vector meson $V = J/\psi , \Upsilon$. (Right) NLO quark contribution. For these graphs all permutations of the parton lines and couplings of the gluon lines to the heavy-quark pair are to be understood. The NLO gluon contribution, with coefficient function $C_g^{\textrm{NLO}}$ and GPD $F_g$, dominates the NLO quark contribution. In these diagrams, the momentum $P\equiv (p+p^\prime )/2$ and *l* is the loop momentum. Note that the momentum fractions of the left and right partons are $x=X+\xi$ and $x'=X-\xi$ respectively; for the gluons connected to the heavy quark-antiquark pair, we have $x' \ll x$ and so $x\simeq 2\xi$

Unfortunately it is not easy to include these data in global parton analyses. The cross section for exclusive meson production is driven not by the usual (diagonal) PDFs but by the more complicated (skewed) generalised parton distributions (GPDs), and is proportional to the skewed gluon-density squared, as indicated in Fig. 1; see [7] for a review. Note from the caption of the figure that $x \approx 2\xi$ at very small *x*, where $2\xi$ is the proton momentum fraction transferred through the GPD to the vector meson.

However, in the small *x* region of our interest, the value of the GPD can be calculated from the conventional PDF with good ($\sim O(x)$) accuracy using the Shuvaev transform [8, 9]. The Shuvaev transform makes use of the fact that as $\xi \rightarrow 0$ (and transverse-momentum transfer $p_T = 0$) the Gegenbauer moments^1^ of the GPD become equal to the known Mellin moments of the PDF. Due to the polynomial condition (see e.g. [11]) even for $\xi \ne 0$ the Gegenbauer moments can be obtained from the Mellin moments to $O(\xi )$ accuracy. Thus it is possible to obtain the full GPD function at small $\xi$ from its known moments.

In principle, this allows us to include the low-*x* exclusive $J/\psi$ data in the global PDF analysis at NLO. In practice the problem is that already at NLO the full Shuvaev transform amounts to a slowly convergent double integral, and the corresponding computation is time consuming as the integrand depends on the derivative of a PDF obtained through interpolation on a grid. This is troublesome for the global fit as, after every iteration, the derivative of the PDF varies and so a new grid defined over the variables $X, \xi$ and $Q^2$ has to be computed to obtain the updated theory prediction. That is, after every iteration, the functional form of the PDF evolves, necessitating the computation of a new GPD grid using the Shuvaev transform. Since the functional forms of PDFs depend on various parameters that can all vary, it is impractical to precompute a grid exhaustively sampling a vast parameter space for every possible form. Therefore, an iterative computation is essential. It may be possible to employ a fast-gridding approach similar to that used for computing inclusive cross sections, e.g. as incorporated in FastNLO [12], APPLgrid [13] or PineAPPL [14], but here we recall the dependence on the PDF for exclusive observables is at the level of the amplitude rather than the total cross section, and so such an approach is less tractable.

In the present paper we describe a method which can overcome this problem by replacing the exclusive vector-meson data at small *x* by ‘effective gluon points’. To demonstrate the efficiency of the proposed method and to show how the inclusion of the LHCb $J/\psi$ and $\Upsilon$ exclusive cross sections affect the result we compare the standard xFitter [15] NLO parton analysis of DIS data with that supplemented by the vector-meson data. Note that our goal is not to present a new set of parton distributions but to emphasise the possible role of the vector-meson data in global parton analyses. The new ‘effective gluon points’ presented in Tables 1 and 2 can be used in future global analyses.

## Effective low-*x* gluon PDF data

To overcome this difficulty, we propose the following procedure. We translate the experimental $J/\psi$ cross section data into a set of “effective” values for the gluon PDF.

**Fig. 2:**
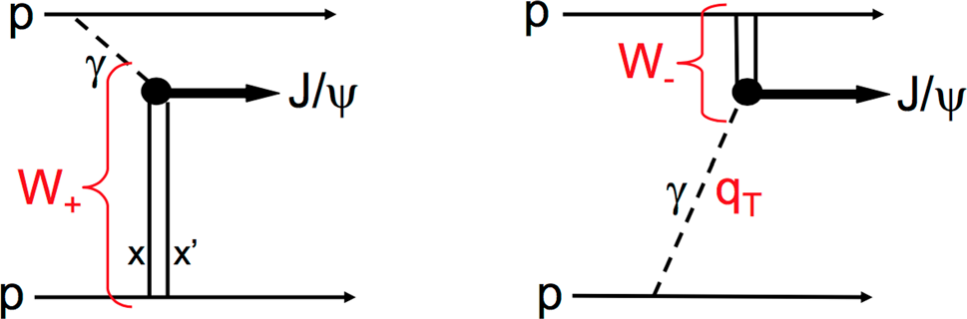
The two diagrams describing exclusive $J/\psi$ production at the LHC. The vertical lines represent two-gluon exchange. The left diagram, the $W_+$ component, is the major contribution to the $pp \rightarrow p+J/\psi +p$ cross section for a $J/\psi$ produced at large rapidity *Y*. Thus such data allow a probe of very low *x* values, $x\sim M_{J/\psi }\,\textrm{exp}(-Y)/\sqrt{s}\,$, where $\sqrt{s}$ is the centre-of-mass energy of the *pp* system; recall that for two-gluon exchange we have $x\gg x'$. The $q_T$ of the photon is very small and so the photon can be considered as a real on-mass-shell particle

**Fig. 3:**
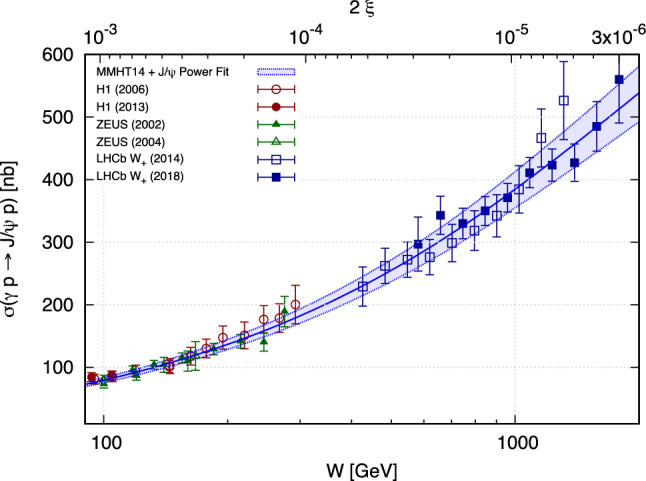
The description of the $J/\psi$ photoproduction HERA [18–21] and LHCb [4, 5] data based on using the central value of the global gluon PDF from the MMHT14 global analysis for $x>10^{-3}$ and a fitted power-like gluon PDF, see Eq. (3), for $x<10^{-3}$. The blue solid line and shaded region show, respectively, the central prediction and propagation of the $\pm \,1 \sigma$ errors on the fitted parameters to the cross-section level

As seen from Fig. 2, the cross section for the exclusive process

1 $$\begin{aligned} p+p\rightarrow p+ J/\psi +p \end{aligned}$$

contains two components,

2 $$\begin{aligned}&\frac{d\sigma ^{(pp \rightarrow p+J/\psi +p)}}{dY}~ \nonumber \\&\quad = ~S^2(W_+)\left( k_+\frac{dn}{dk_+}\right) \sigma _+(\gamma p \rightarrow J/\psi p)~+~W_-~\textrm{term}, \end{aligned}$$

corresponding to energies $W_{\pm }$, where $W_+$ denotes the energy when the vector meson travels in the same direction as the photon, and $W_-$ when it travels in the opposite direction. We see that the values of the subprocess cross sections $\sigma (\gamma p \rightarrow J/\psi p)$, for each rapidity *Y* of the $J/\psi$, are weighted by the corresponding gap survival factors $S^2(W_\pm )$ [16] and photon fluxes $dn/dk_\pm$ [17]. The cross section with the lower $\gamma p$-energy ($W_-$) was measured at HERA [18–21] and can be calculated with sufficient accuracy since it corresponds to relatively large *x* where the uncertainties in the parton distributions are small. Hence the exclusive $J/\psi$ data provide reliable values for $\sigma _+(\gamma + p\rightarrow J/\psi +p)$, corresponding to the component with the larger $\gamma p$-energy, $W_+$, see Fig. 3.^2^

These $\sigma _+$ data were well described in [22] by a power-like low-*x* gluon

3 $$\begin{aligned} xg(x)=A\cdot x^{-\lambda } ~~~~~ \textrm{with} ~~~~~ \lambda =0.135\pm 0.006, \end{aligned}$$

also shown in Fig. 3. This provides the initialisation values for $xg_\textrm{fit}(x)$, described later.

In collinear factorisation at NLO accuracy, the amplitude for the exclusive photoproduction of heavy quarkonium off unpolarised protons, $\gamma p \rightarrow V p$, has the form

4 $$\begin{aligned}&\mathcal{M}(\gamma +p\rightarrow V+p)\nonumber \\  &\quad =\sum _{i=g,q} N_i\int _{-1}^1 \frac{dX}{X} ~F_i(X,\xi ) ~C^{\textrm{NLO}}_{i}(\xi /X)\ , \end{aligned}$$

where the constants $N_i$ provide the correct normalisation, see e.g. [22], and $F_i$ and $C_i$ denote quark and gluon GPDs and coefficient functions respectively. Additional dependences on the renormalisation and factorisation scales, and the four-momentum transfer $t=t_\textrm{min}=(2\xi m_p)^2/(1-\xi ^2)\sim 0$, are not shown explicitly in Eq. (4).

We note here the sensitivity to the PDF or GPD at the *amplitude* level. In our approach, the small $\xi$ GPD is obtained from the PDF using the Shuvaev transform [8, 9]

5 $$\begin{aligned} F_q(X, \xi )= &   \sqrt{1-\xi ^2}\int _{-1}^1dx'~ \biggl [ \frac{2}{\pi } \text {Im} \nonumber \\  &   \quad \times \int _0^1 \frac{ds}{y(s) \sqrt{1-y(s)x'}} \biggr ] \frac{d}{dx'} \left( \frac{q(x')}{|x'|} \right) ,\nonumber \\ F_g(X, \xi )= &   \sqrt{1-\xi ^2} \int _{-1}^1dx'~ \nonumber \\  &   \quad \times \biggl [ \frac{2}{\pi } \text {Im} \int _0^1 \frac{d s~(X+\xi (1-2s)}{y(s) \sqrt{1-y(s)x'}} \biggr ] \nonumber \\    &   \quad \frac{d}{dx'} \left( \frac{g(x')}{|x'|} \right) , \end{aligned}$$

where the kernel of the transform,

6 $$\begin{aligned} y(s) = \frac{4s(1-s)}{X+\xi (1-2s)}. \end{aligned}$$

The subprocess cross sections

7 $$\begin{aligned}&\sigma (\gamma +p \rightarrow V+p) = \frac{1}{B(W)} \frac{ d \sigma }{dt}\bigg |_{t=0}\,,\,\,\,\,\,\text {where}\,\,\,\,\,\,\nonumber \\  &\frac{d \sigma }{dt}\bigg |_{t=0} = \frac{|\mathcal M|^2}{16 \pi W^4}, \end{aligned}$$

and *B*-slope $B(W)\,\textrm{GeV}^2= B_0 + 4\alpha _{{P}}' \ln (W/90)$. The intercept $B_0 = 4.9~(4.63)$ for $J/\psi$ ($\Upsilon$) production and $\alpha _{P}' = 0.06$ [23].

Moreover it was shown that after the optimal factorisation scale,^3^$\mu _F = M_{J/\psi }/2$, was chosen, where $M_{J/\psi }$ is the mass of the $J/\psi$, and the so-called $Q_0$ subtraction [26]^4^ was performed, the quark contribution to $\sigma _+$ becomes negligible [28]. That is the value of

8 $$\begin{aligned} \sigma _+ \,\propto \,(\,2\xi \, g(2\xi )\,)^2 \end{aligned}$$

is just proportional to the gluon density squared.^5^

The imaginary part of the LO coefficient function obtained through the on-shell Cutkosky-cut of the LO Feynman diagrams (an example of which is shown in Fig. 1), enforces $X=\xi$. At NLO, this is no longer true and there is a smearing effect (i.e. a tail around $X=\xi$), but the integrand remains highly-peaked around this point, particularly for the gluon coefficient function [28]. In principle, while each value of the skewness parameter $\xi$ entails an integration over *X*, the dominant contribution comes from $X \approx \xi$ and so for each experimental data point at a given photon-proton centre-of-mass energy *W*, we can associate a skewness $\xi \approx M_{J/\psi }^2/(2W^2)$ and the value $x=X+\xi \approx 2\xi$. It is in this sense that Eq. (8) should be understood.

Now for each data point, *i*, we calculate the predicted $J/\psi$ cross section $\sigma _+(\textrm{fit})_i$ using the gluon GPDs obtained via the Shuvaev transform of our gluon PDF fit in Eq. (3) and compare it to the experimental value $\sigma _+(\textrm{data})_i$.^6^ The ratio $\sigma _+(\textrm{data})_i/\sigma _+(\textrm{fit})_i$ is approximately equal to the square of the ratio of the gluon densities9In other words, in this way we can calculate the effective gluon density (PDF) corresponding to $x_i=2\xi _i$ as

10 $$\begin{aligned} g_{\textrm{eff}}(x_i,\mu _{\textrm{opt}})~~=~~g_{\textrm{fit}}(x_i,\mu _{\textrm{opt}})~~\sqrt{\frac{\sigma _+{(\textrm{data})_i}}{\sigma _+(\textrm{fit})_i}} . \end{aligned}$$

Correspondingly the error11The error of the lower-energy contribution, $\sigma _-(x_i)$, is accounted for in the experimental value of $\sigma _+(\textrm{data})_i$. Recall that this lower-energy contribution is small and its errors are negligible.

**Table 1:** The starting values of $xg_\textrm{fit}$ and the final values of $xg_\textrm{eff}$ and $\delta xg_\textrm{eff}$ obtained from eqns. (10) and (11) at $Q^2 = M_{J/\psi }^2/4 = 2.4\,\textrm{GeV}^2$ using the exclusive $J/\psi$ production data at 7 TeV [4] and 13 TeV [5] obtained by the LHCb Collaboration

**13 TeV**	*x*	$xg_\textrm{fit}$	$xg_\textrm{eff} {\pm } \delta xg_\textrm{eff}$	**7 TeV**	*x*	$xg_\textrm{fit}$	$xg_\textrm{eff} {\pm } \delta xg_\textrm{eff}$
	$2.84 \times 10^{-5}$	3.71	3.83 ${\pm }$ 0.26		$5.28 \times 10^{-5}$	3.41	3.45 ${\pm }$ 0.24
	$2.21 \times 10^{-5}$	3.83	4.08 ${\pm }$ 0.17		$4.11 \times 10^{-5}$	3.52	3.65 ${\pm }$ 0.20
	$1.72 \times 10^{-5}$	3.97	3.96 ${\pm }$ 0.14		$3.20 \times 10^{-5}$	3.64	3.68 ${\pm }$ 0.19
	$1.34 \times 10^{-5}$	4.10	4.05 ${\pm }$ 0.13		$2.49 \times 10^{-5}$	3.77	3.67 ${\pm }$ 0.19
	$1.04 \times 10^{-5}$	4.25	4.14 ${\pm }$ 0.12		$1.94 \times 10^{-5}$	3.90	3.79 ${\pm }$ 0.19
	$8.14 \times 10^{-6}$	4.39	4.32 ${\pm }$ 0.13		$1.51 \times 10^{-5}$	4.04	3.88 ${\pm }$ 0.19
	$6.34 \times 10^{-6}$	4.54	4.35 ${\pm }$ 0.13		$1.18 \times 10^{-5}$	4.18	3.99 ${\pm }$ 0.20
	$4.93 \times 10^{-6}$	4.70	4.34 ${\pm }$ 0.14		$9.18 \times 10^{-6}$	4.32	4.19 ${\pm }$ 0.20
	$3.84 \times 10^{-6}$	4.86	4.60 ${\pm }$ 0.17		$7.15 \times 10^{-6}$	4.47	4.59 ${\pm }$ 0.23
	$2.99 \times 10^{-6}$	5.03	4.91 ${\pm }$ 0.27		$5.57 \times 10^{-6}$	4.62	4.84 ${\pm }$ 0.28

We therefore propose to include these “effective” gluon PDF data points calculated according to Eqs. (10) and (11) in a parton fit analysis in order to reduce the present uncertainties in the behaviour of the low-*x* partons. Since the final parton distribution may differ from Eq. (3) obtained from [22], to achieve greater accuracy in the final determined values of $xg_\textrm{eff}$, several additional iterations in the $xg_\textrm{eff}$ data may be needed. We therefore proceed as follows. We first calculate the cross section $\sigma _{+}(\textrm{fit})_i$ using the values of $x_ig_\textrm{fit}(x_i)$ in Eq. (3) to obtain starting values of $x_ig_\textrm{eff}(x_i)$. Such values are then used in our parton fit analysis, however they may not necessarily be sufficiently accurate to provide to the community for use in other global fits. Consequently, the outputted, new gluon PDFs $xg_\textrm{fit}$ from our parton fit analysis are then passed to our Shuvaev-integral transform routine to obtain updated GPDs via Eq. (5) and subprocess cross sections $\sigma _+(\textrm{fit})$ via Eq. (7). These are then used to re-calculate $xg_\textrm{eff}$ via Eq. (10) and used in a second parton fit analysis. The procedure continues until the resulting $xg_\textrm{eff}$ values from parton analysis *n* do not differ from those in analysis $n-1$ by $\pm 1 \sigma$. Fortunately, such a procedure is well convergent. The final determined *iterated* data points are given in Table 1.^7^

In Table 2, we show the final effective iterated gluon points for exclusive $\Upsilon$ production [6] at the corresponding ‘optimal’ scale 22.4 GeV$^2$. The accuracy of these points is worse than that obtained from $J/\psi$ production, but they allow for a constraint on the $Q^2$-evolution in the low-*x* region. So far the exclusive $\Upsilon$ data for the $\gamma p \rightarrow \Upsilon p$ cross section from the LHCb Collaboration have been obtained using gap survival factors $S^2(W_{\pm })$ and photon fluxes $dn/dk_{\pm }$ that need updating or correcting, see [29]. Nevertheless, the resulting $\Upsilon$ data shown in Table 2 already include the corresponding corrections, which however never exceed the experimental error bars. Note that the resulting $\Upsilon$ points already show evidence of a rising gluon PDF power behaviour in the region $10^{-5}< x < 10^{-4}$ and $Q^2 = m_b^2 \approx 22~\textrm{GeV}^2$ (see Fig. 2 of [29]). Recall that as was shown in [29], at the border of the LHCb acceptance, the choice of survival factor and photon flux only amounted to 5% for $J/\psi$ production, while for $\Upsilon$ production it is of the order of 25%.

## The impact of the low-*x* gluon effective data points on a global analysis

**Fig. 4:**
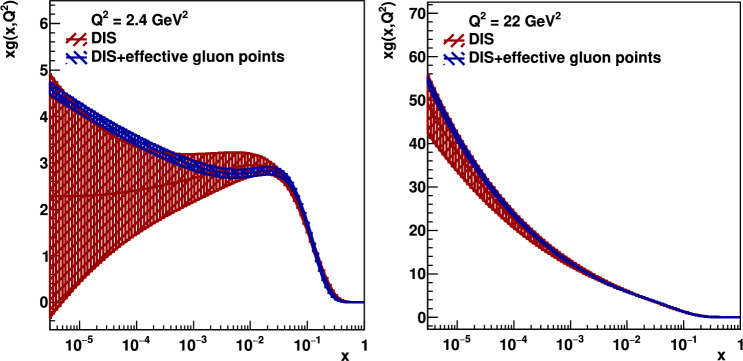
The gluon distributions *xg* at scales $\mu _F^2 = Q^2 =$ 2.4 GeV$^2$ (left) and 22 GeV$^2$ (right) obtained in xFitter fitting DIS (red) and DIS+effective gluon data points (blue)

The gluon parton distribution resulting from a global xFitter [15] NLO analysis of pure Deep-Inelastic-Scattering (DIS) data (red) and DIS data supplemented with the effective gluon points extracted from the exclusive $J/\psi$ and $\Upsilon$ production at the LHC (blue) are shown in Fig. 4. Here we have used the standard xFitter ensemble of DIS data and the standard xFitter ansatz for the input parton parametrizations with the kinematic cut $Q^2 > 2.4$ GeV$^2$. The datasets used and the partial and total minimum $\chi ^2$ figure of merit per degree of freedom, $\chi _{\text {min}}^2/\text {d.o.f}$, for both fits are shown in Table 3.

**Table 2:** The starting values of $xg_\textrm{fit}$ and the final values of $xg_\textrm{eff}$ and $\delta xg_\textrm{eff}$ obtained from eqns. (10) and (11) at $Q^2 = M_{\Upsilon }^2/4 = 22.4\,\textrm{GeV}^2$ using the exclusive $\Upsilon$ production data at 7, 8 TeV [6] obtained by the LHCb Collaboration

	**7, 8 TeV**	*x*	$xg_\textrm{fit}$	$xg_\textrm{eff} \pm \delta xg_\textrm{eff}$
		$1.01 \times 10^{-4}$	23.5	19.9 ${\pm }$ 3.1
		$4.77 \times 10^{-5}$	28.3	23.9 ${\pm }$ 3.3
		$2.26 \times 10^{-5}$	34.1	31.1 ${\pm }$ 4.1

**Fig. 5:**
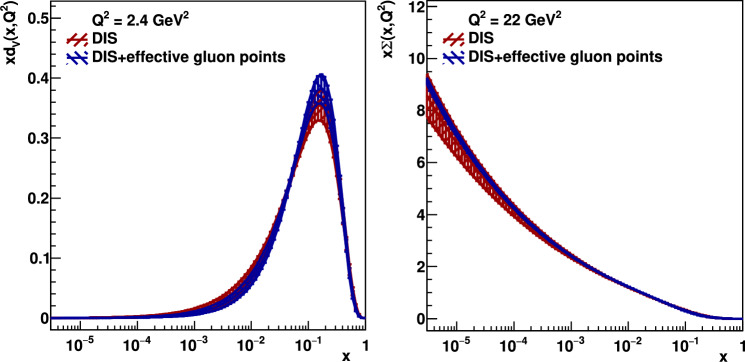
The valence $xd_v$ quark distribution at scale 2.4 GeV$^2$ (left) and the singlet $x\Sigma$ quark distribution at 22 GeV$^2$ (right) obtained in xFitter fitting DIS data (red) and DIS + effective gluon data points (blue)

**Fig. 6:**
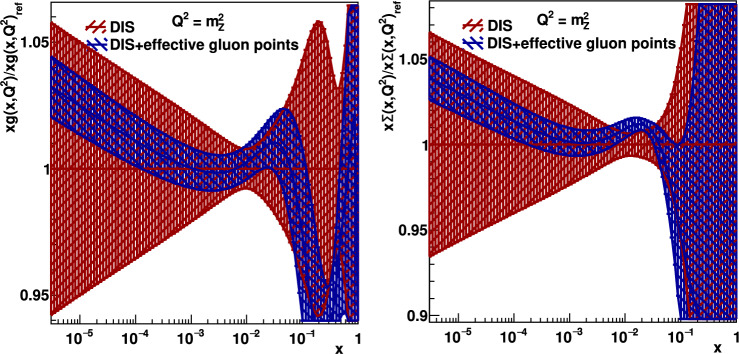
The ratio of the fitted ‘DIS + effective gluon points’ gluon *xg* (left) and quark singlet $x\Sigma$ (right) PDFs to the DIS-only ones, $xg_\textrm{ref}$ and $x\Sigma _\textrm{ref}$, respectively, at $Q^2=M_Z^2$

It is clearly seen in Fig. 4 that the inclusion of the low-*x* effective gluon points extracted from the exclusive heavy vector-meson production at the LHC essentially improve the accuracy of the global parton analysis, and we obtain a good overall final $\chi _{\text {min}}^2/\text {d.o.f} \sim O(1)$. The blue error band is much smaller than the red one. Moreover, the inclusion of new information obtained from the heavy vector-meson ultraperipheral production affects (albeit expectedly not so strongly) even the quark distribution as demonstrated, for example, in Fig. 5. The new $g_{\text {eff}}$ low-*x* points affect the quark distribution even at a rather large *x* due to the energy-momentum sum rules and the particular choice of the fixed form of the input ansatz. In Fig. 6, we show the ratio of our fitted ‘DIS + effective gluon points’ gluon and quark singlet PDFs to the central value of the DIS-only ones at a much larger scale, $Q^2 = M_Z^2$. Since during the $Q^2$ evolution the value of *x* decreases, the parton densities at such large $Q^2$ originate mainly from the relatively large-*x* input PDF, where the distribution is already well fixed by DIS data. Therefore, the effect of adding the new $J/\psi$ data is less pronounced at $Q^2=M^2_Z$, but still visible, as shown.

As usual, additional theoretical uncertainties such as the correction to the Non-Relativistic QCD (NRQCD) approach used for the $q\bar{q} \rightarrow V$ transition in the computation of $\sigma (\gamma p\rightarrow V+p)$, or possible higher-twist effects, or effects of absorptive corrections, or other higher-order corrections, are not shown. Note that, apart from the absorptive effects, all other corrections (e.g. NRQCD) do not affect the energy behaviour of the amplitude, but just change its normalisation. Indeed, to ‘know’ the initial energy we need an additional gluon, which connects the upper (photon block in Fig. 1) with the lower/target parts of the diagram. This would correspond to a higher-twist contribution and should be suppressed by a large factorisation scale $\mu _F$. Typically, these corrections act ‘locally’ in rapidity space and therefore do not affect the energy behaviour of the amplitude, only its normalisation. On the other hand, our approach, in its present form, satisfactorily describes the available HERA data [22]. Recall that, as was shown in [30], normalising the vector-meson wave function to the experimentally measured $J/\psi \rightarrow l^+l^-$ decay width allows us to account for relativistic corrections in the NRQCD approach with good accuracy (within a few percent).

**Table 3:** The partial $\chi _{\text {min}}^2/\text {d.o.f}$ for each dataset included in the baseline fit (DIS) and with the effective gluon data points added (DIS + eff. gluon pts.). Here, we use the DIS data in the kinematic range $Q^2 > 2.4$ GeV$^2$. The total $\chi _{\text {min}}^2/\text {d.o.f}$ is also given

Dataset	$\chi _{\text {min}}^2/\text {d.o.f}$ (DIS)	$\chi _{\text {min}}^2/\text {d.o.f}$ (DIS+eff. gluon pts.)
HERA1+2 NCep 820	80/73	79/73
HERA1+2 NCep 460	220/207	220/207
HERA1+2 CCep	43/39	44/39
HERA1+2 NCem	221/159	220/159
HERA1+2 CCem	54/42	56/42
HERA1+2 NCep 575	223/257	227/257
HERA1+2 NCep 920	465/391	470/391
LHC excl. $J/\psi$ *pp* 7 TeV	N/A	8.95/10
LHC excl. $J/\psi$ *pp* 13 TeV	N/A	3.51/10
LHC excl. $\Upsilon$ *pp* 7,8 TeV	N/A	3.23/3
Total $\chi _{\text {min}}^2/\text {d.o.f}$	1412/1154 $\approx$ 1.22	1444/1177 $\approx$ 1.23

## Working to NNLO via a *K* factor

In principle, the method of “effective gluon points” described above can be extended to NNLO. Unfortunately at present the coefficient functions for $J/\psi$ and $\Upsilon$ photo- and electroproduction are known only at NLO level [27, 31, 32].

However, it is possible to include these points in a NNLO global analysis in an approximate way by introducing a NNLO/NLO *K* factor, whose value can be extracted from the description of existing HERA $\gamma +p\rightarrow J/\psi +p$ (and/or $\gamma +p\rightarrow \Upsilon +p$) data. In the following, we denote this analysis NNLO* to distinguish it from an analysis in which the full NNLO coefficient functions would be used when they become available. In general the *K* factor may depend on the factorisation scale and on the ratio $z=(X+\xi )/2\xi$ of the parton momentum fractions $x=(X+\xi )$ to $\xi$. That is the *K* factor should be included in the convolution for the $\gamma +p\rightarrow J/\psi +p$ amplitude

12 $$\begin{aligned}&\mathcal{M}(\gamma +p\rightarrow J/\psi +p)~~\nonumber \\&\quad =~~\sum _{i=g,q} N_i\int _{-1}^1 \frac{dX}{X} ~F_i(X,\xi )~K ~C^{\textrm{NLO}}_{i}(\xi /X)\ , \end{aligned}$$

given previously. The integral in Eq. (12) converges^8^ at $X\sim \xi$. This means that actually after the convolution of the coefficient function with the gluon distribution the effective NNLO/NLO *K* factor may be considered as a constant *assuming that the power *$\lambda$ *of Eq.* (3) *does not vary in the interval of interest*.

Thus the value of this *K* factor can be estimated by describing the exclusive $J/\psi$ photoproduction data from HERA with the NNLO partons and NLO coefficient functions in the region $0.01>x>0.001$ since here the uncertainties in the gluon distribution given by the present global analysis are relatively small. In other words the square of the *K* factor can be obtained as the ratio of the measured $\gamma +p\rightarrow J/\psi +p$ cross section, $\sigma (\textrm{data})$, to $\sigma ^{\textrm{NLO}/\textrm{NNLO}}$ calculated using the NLO coefficient function but NNLO input partons (PDF/GPD). That is,

13 $$\begin{aligned} K_i=\sqrt{\left( \frac{\sigma (\textrm{data})}{\sigma ^{\textrm{NLO}/\textrm{NNLO}}}\right) _i}\,, \end{aligned}$$

and then make the trivial average of the *K* factors for each data point. From Fig. 3 we may hope that the power $\lambda$ which describes the energy behaviour of the $\gamma +p\rightarrow J/\psi +p$ cross section is more or less constant. However, as it is seen from Eq. (12), this would require the pure power behaviour for the gluons and not for the $J/\psi$ cross section. Unfortunately in the present analysis just in the region of interest $0.01>x>0.001$ we observe that the gluon PDF is too flat (see Fig. 4 left). The power growth starts only at $x<0.001$, while it is well known that the $J/\psi$ photoproduction data from HERA are all well described by a power behaviour starting at $x < 0.01$ [19, 21]. Most probably this is explained by the fact that the DGLAP evolution does not account for the (higher twist) absorptive corrections which are not negligible in this low-*x* region. To mimic the role of these corrections in DIS data, the fit chooses gluons which slightly decrease at $x<0.01$.

In a future analysis, we have two possibilities. Either to include these absorptive effects into the DGLAP evolution following the GLR-MQ scheme [33, 34] (with the possibility that this will generate power increasing gluons already in the HERA domain, $x<0.01$), or to have at hand the full NNLO coefficient function to reach complete NNLO accuracy and go beyond the NNLO* approach discussed here.

In any case in order to obtain realistic partons at such low *x* and scales, the absorptive effect should be accounted for.

## Conclusion and outlook

We have described the method of “effective gluon points” which readily allows for the inclusion of exclusive heavy vector-meson *V* photoproduction data, where $V = J/\psi , \Upsilon$ (as well as the exclusive production in ultraperipheral events at the LHC) in a conventional global parton analysis. Using xFitter, we fit DIS data together with these effective gluon points extracted from the $J/\psi$ and $\Upsilon$ exclusive production data from LHCb and demonstrate that this crucially improves the accuracy of the obtained gluon distribution in the low-*x* domain. The values of $xg_\textrm{eff}(x)$ extracted from the available exclusive $J/\psi$ and $\Upsilon$ data from LHCb are presented in Tables 1 and 2.

Since at present the NNLO coefficient functions for the photon to vector meson ($\gamma \rightarrow V$) transition are not available, our computations were performed at NLO level. However, there is the possibility to reach NNLO* accuracy, by extracting the NNLO/NLO *K* factor from the existing HERA $\gamma +p\rightarrow J/\psi +p$ data as discussed in Sect. 4. Moreover, exclusive vector-meson production data have already been collected by the LHC and more should follow. This merits further theoretical study of the NNLO* analysis, which we reserve for future work.

The analysis presented in this work has demonstrated the potential of existing exclusive data to significantly improve the NLO global parton distributions at small *x*.

## Data Availability

Data will be made available on reasonable request. [Author’s comment: The datasets generated during and/or analysed during the current study are available from the corresponding author on reasonable request.]

## Footnotes

1 Gegenbauer moments are the analogue of Mellin moments which diagonalize the $Q^2$ evolution of PDFs. The corresponding operator diagonalizes the $Q^2$ evolution of the GPDs [10]. As $\xi \rightarrow 0$ the Gegenbauer moments become equal to the Mellin moments.

2 Actually, the subtraction of the $W_-$ contribution was done already in the LHCb analyses where the results are presented as the $\sigma _+(\gamma + p \rightarrow V+p)$ cross sections.

3 At this optimal scale the large double-logarithmic contributions of the form $c_n(\alpha _s\ln (1/\xi )\ln \mu _F^2)^n$ are resummed into the incoming PDF/GPD [24, 25].

4 The $Q_0$ subtraction is necessary to exclude the double counting between the low $l_T$ part of the coefficient function and the PDF-*input*. This, together with the resummation of the aforementioned double-logarithmic terms [24, 25], helps mitigate the problem of the strong factorisation scale dependence [27] of the NLO amplitude.

5 Strictly speaking, the value of $\sigma _+$ is proportional to the square of the corresponding GPD.

6 For the results presented later in Tables 1 and 2, the Shuvaev transform is used in the region $x \le 10^{-4}$.

7 Note that we do not fit to the individual $\sigma (\gamma +p\rightarrow J/\psi +p)$ data points in our approach. Instead, we use effective $xg_{\textrm{eff}}(x)$ points. Recall the values of $xg_\textrm{fit}(x)$ were obtained from a previous fit to the $J/\psi$ cross section only, and thus may not be sufficiently accurate for a global analysis. Therefore we do not use the values of $xg_\textrm{fit}(x)$ from this former $J/\psi$ fit, but instead fit to the corrected $g_{\textrm{eff}}(x)=g_{\textrm{fit}}(x)\sqrt{\sigma (\textrm{data})(x)/\sigma (\mathrm{{fit})}(x)}$ data points. These effective values will provide a better overall description of the data and a better stability, so that the true values will not be changed too much in the fit iteration.

8 It is known that the $\gamma +p\rightarrow J/\psi +p$ amplitude increases as a power of the photon energy (i.e. as a power of $1/\xi$). On the other hand the NNLO gluon coefficient function can contain only $\ln ^2z$ from the higher-order gluon loop insertions. Thus at large energy (i.e. very small $\xi$) the dominant contribution to (12) comes from $z\sim O(1)$ (i.e. $X\sim \xi$) while the power growth of the amplitude is provided by the GPD power behaviour. In this case we have to calculate the $\gamma +p\rightarrow J/\psi +p$ NNLO amplitude, $\mathcal{M}$, by multiplying the NLO coefficient functions, $C_i{^\textrm{NLO}}$ with $i=g,q$, by this *K* factor.

## Code Availability Statement

Code/software will be made available on reasonable request. [Author’s comment: The code/software generated during and/or analysed during the current study is available from the corresponding author on reasonable request.]
